# Nrf2 epigenetic derepression induced by running exercise protects against osteoporosis

**DOI:** 10.1038/s41413-020-00128-8

**Published:** 2021-02-26

**Authors:** Xingren Chen, Xiaobo Zhu, Ai Wei, Fang Chen, Qi Gao, Ke Lu, Qing Jiang, Wangsen Cao

**Affiliations:** 1 Department of Sports Medicine and Adult Reconstructive Surgery, State Key Laboratory of Pharmaceutical Biotechnology, The Affiliated Nanjing Drum Tower Hospital of Nanjing University Medical School, Nanjing, 210008 China; 2 Center for Organ Fibrosis and Remodeling Research, Jiangsu Key Lab of Molecular Medicine, Nanjing University Medical School, Nanjing, 210093 China

**Keywords:** Pathogenesis, Bone quality and biomechanics

## Abstract

Osteoporosis (OP) is a common skeletal disease involving low bone mineral density (BMD) that often leads to fragility fracture, and its development is affected by multiple cellular pathologies and associated with marked epigenetic alterations of osteogenic genes. Proper physical exercise is beneficial for bone health and OP and reportedly possesses epigenetic modulating capacities; however, whether the protective effects of exercise on OP involve epigenetic mechanisms is unclear. Here, we report that epigenetic derepression of nuclear factor erythroid derived 2-related factor-2 (Nrf2), a master regulator of oxidative stress critically involved in the pathogenesis of OP, mediates the significant osteoprotective effects of running exercise (RE) in a mouse model of OP induced by ovariectomy. We showed that Nrf2 gene knockout (Nfe2l2^−/−^) ovariectomized mice displayed a worse BMD reduction than the controls, identifying Nrf2 as a critical antiosteoporotic factor. Further, femoral Nrf2 was markedly repressed with concomitant DNA methyltransferase (Dnmt) 1/Dnmt3a/Dnmt3b elevations and Nrf2 promoter hypermethylation in both patients with OP and ovariectomized mice. However, daily 1-h treadmill RE significantly corrected epigenetic alterations, recovered Nrf2 loss and improved the femur bone mass and trabecular microstructure. Consistently, RE also normalized the adverse expression of major osteogenic factors, including osteoblast/osteoclast markers, Nrf2 downstream antioxidant enzymes and proinflammatory cytokines. More importantly, the RE-conferred osteoprotective effects observed in the wild-type control mice were largely abolished in the Nfe2l2^−/^^−^ mice. Thus, Nrf2 repression due to aberrant Dnmt elevation and subsequent Nrf2 promoter hypermethylation is likely an important epigenetic feature of the pathogenesis of OP, and Nrf2 derepression is essential for the antiosteoporotic effects of RE.

## Introduction

Osteoporosis (OP) is a progressive skeletal disease involving low bone mineral density (BMD) that often affects the aged population, especially postmenopausal women, and potentially leads to fragility fracture.^1^ To maintain proper strength and integrity, bone undergoes constant turnover that is controlled by a balance between osteoclast-maintained bone resorption (osteoclastogenesis) and osteoblast-induced bone formation (osteoblastogenesis).^2^ OP is a result when osteoclastogenesis exceeds osteoblastogenesis and is histomorphologically characterized by reduced BMD and trabecular microstructure deterioration. The pathogenesis of OP involves various endogenous and exogenous factors, including aging, weight-related mechanical stimulation, abnormal mineral and hormone metabolism, and stresses of various natures that are mechanistically linked to the altered transcription of osteogenic genes. Recent studies have shown that abnormal epigenetic modification participates in the control of osteoclastogenesis and osteoblastogenesis.^3,4^ In particular, DNA methylation profiling investigations detect many genomic loci/genes that are modified by DNA methylation alterations in the trabeculae of patients with OP,^5,6^ identifying another fundamental mechanism of the pathogenesis of OP.

DNA methylation is one of the most stable epigenetic modifications of gene transcription, affects nearly 60%–90% of genomic CpG dinucleotides^7^ and is inversely regulated by two groups of enzymes with opposite catalytic functions. Three bioactive DNA methylation transferases (Dnmt), namely, maintenance Dnmt1 and de novo Dnmt3a and Dnmt3b, catalyze DNA methylation by adding a methyl group (CH_3_) to the cytosine of CpG dinucleotides enriched in gene promoters/enhancers (CpG islands) that block transcription factor access and silence gene transcription. In contrast, DNA demethylation is mediated by ten-eleven translocation family enzymes via sequential oxidization of 5mC–5hmC and its intermediates and completed by a base excision process.^7^ DNA methylation is reversible, and DNA demethylating drugs have shown therapeutic benefits against various chronic and aging-related disorders, including OP, in animal studies;^8,9^ however, their long-term use might cause intolerable side effects in certain patients.^10^ Recent studies have discovered that proper physical exercises can beneficially modulate DNA methylation in various physiological and pathological conditions, including aging, cancer, and type II diabetes.^11–16^ Physical exercises are feasible, free of side effects, beneficial for maintaining bone health and prophylactic against OP;^17,18^ however, whether their antiosteoporotic effects involve epigenetic mechanisms and the critical genes mediating this protection are intriguing but not experimentally established.

Weak oxidative stress responses and the associated low circulating antioxidants causally relate to lower BMD,^19,20^ suggesting that redox imbalance is a risk factor of OP. Nrf2 (nuclear factor erythroid derived 2-related factor-2) is a transcription factor essential for host cellular defense against oxidative stress and bone homeostasis.^21^ Nrf2 is ubiquitously expressed in almost all cell types, including osteoblasts, osteocytes, and osteoclasts, in an inactive form in the cytoplasm. Upon stimulation by stress of various natures, Nrf2 is released from its inhibitor Keap1 and translocates to the cell nucleus, where it binds to the antioxidant-response element on target gene promoters and transactivates multiple antioxidant and detoxification enzymes, such as catalase, superoxide dismutase (SOD) and glutathione peroxidase (GPX).^22^ Nrf2 genetic or pathological deficiencies correlate with increased susceptibility to OP in mice due to an insufficient antioxidative stress capacity,^23,24^ while enhancing Nrf2 by various activators or agonists is protective against OP.^25,26^ The Nrf2 promoter is characterized by CpG islands, and Nrf2 downregulation due to altered DNA methylation is observed in several aging-related pathological conditions, such as aging, cancer and neurodegenerative disease,^27,28^ suggesting that epigenetic Nrf2 suppression might contribute to the initiation or progression of OP.

In this study, we found that reduced femoral Nrf2 due to aberrant DNA methylation modifications is causally involved in OP development. We further demonstrated that running exercise (RE) confers effective osteoprotection against OP by epigenetic Nrf2 recovery in a mouse model of OP induced by ovariectomy (Ovx). These results revealed an important epigenetic mechanism of the pathogenesis of OP and provide a molecular basis for the known osteoprotection of physical exercise against OP.

## Results

### Nrf2 is a critical antiosteoporotic factor suppressed in osteoporotic femurs

**Fig. 1:**
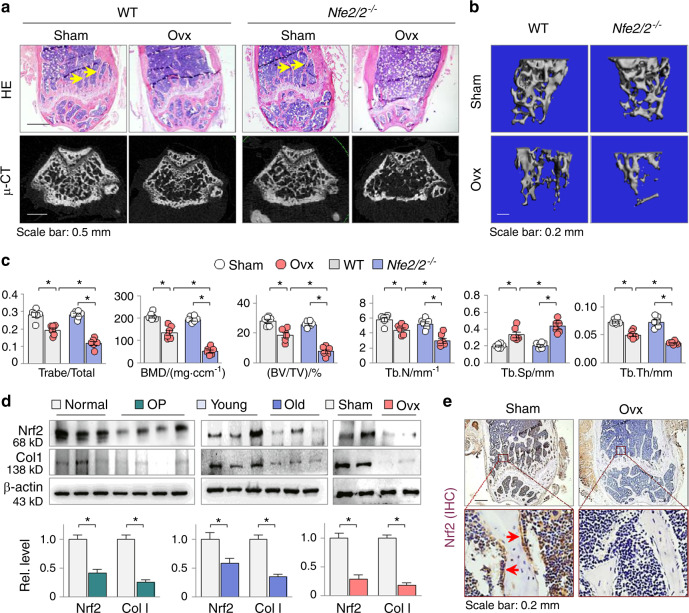
Nrf2 is a critical antiosteoporotic factor suppressed in osteoporotic femurs. Representative photomicrographs of (**a**) H&E-stained (the upper panel, the arrows indicate trabeculae) and micro-CT-scanned (μ-CT, the lower panel) distal femur sections and (**b**) micro-CT 3D trabecular architecture of distal femurs from the Nrf2 knockout mice (*Nfe2l2*^−*/−*^) and wild-type littermates (WT) that underwent sham treatment or ovariectomy (Ovx) for 5 weeks (6 mice in each group). **c** Quantitative analysis of the ratio of sectional trabeculae over total area (Trabe/Total), microstructural bone mineral density (BMD, mg·ccm^−1^), the ratio of bone volume to tissue volume (BV/TV), trabecular number (Tb.N), trabecular thickness (Tb.Th) and trabecular separation (Tb.Sp) of the μ-CT-scanned distal femurs in Fig. 1b. Data are presented as the mean ± SEM. *P < 0.05, two-way ANOVA. **d** Western blot analysis of Nrf2 and collagen 1 (Col 1) from the femoral homogenates of 3 healthy controls/4 patients with OP, sham/ovariectomized female mice, and young (3 months)/old (25 months) male mice (6 mice in each group, three or two randomly selected samples are shown). β-actin served as the internal control. WB quantifications are shown underneath the blots. Relative values are presented as the mean ± SEM of all human and mouse samples. *P < 0.05, one-way ANOVA. **e** Representative femur sections of the sham and ovariectomized mice stained for Nrf2 by immunohistochemistry (IHC). The lower panel images are enlarged frames in the upper panel. The positively stained cells in brown are indicated by arrows

To gain insight into the critical role of Nrf2 in OP development, we adopted a well-known mouse Ovx model of OP.^29^ Female Nrf2 gene knockout mice (*Nfe2l2*^*−/−*^) and wild-type (WT) littermates receiving Ovx surgery for 5 weeks displayed thinned osteoporotic trabeculae with lost continuity and enlarged areolae in the distal femurs (Fig. 1a). To better quantify the osteoporotic changes, we also scanned the femurs by microcomputed tomography (μ-CT, Fig. 1a, the lower panel and Fig. 1b) and found that the ratio of trabeculae to the total area of the *Nfe2l2*^*−/−*^ sham mice was slightly but not significantly reduced compared to that of the WT sham mice (0.281 ± 0.009 for the *Nfe2l2*^*−/−*^ mice versus 0.292 ± 0.006 for the WT sham mice, *P* < 0.05). As anticipated, the WT mice showed a significant reduction in trabeculae over the total area after Ovx (0.192 ± 0.012 for the WT ovariectomized mice vs. 0.292 ± 0.006 for the WT sham mice, *P* < 0.05, Fig. 1a, the lower panel and c), but the *Nfe2l2*^*−/−*^ mice displayed an even worse trabecular reduction (0. 116 ± 0.012 vs. 0.192 ± 0.012 for the Nrf2 WT ovariectomized mice, *P* < 0.05. Fig. 1c). We further analyzed the microstructure of the distal femur and found that the bone parameters of both the *Nfe2l2*^*−/−*^ and WT sham mice were similar, but the *Nfe2l2*^*−/−*^ ovariectomized mice displayed significantly worse BMD, trabecular bone volume versus tissue volume (BV/TV), trabecular bone number (Tb.N), trabecular thickness (Tb.Th) and trabecular bone separation (Tb.Sp) than the control ovariectomized mice (Fig. 1c), confirming that mice lacking Nrf2 show enhanced pathogenesis of OP.^23^ Intriguingly, the femoral levels of Nrf2 and collagen 1, the major organic component of the extracellular matrix in bone, were significantly reduced in all 4 patients with OP, male aging mice (25 months) and female ovariectomized mice (Fig. 1d). Immunohistochemistry (IHC) confirmed that the Nrf2 expression level was markedly reduced in the distal femur of the ovariectomized mice (Fig. 1e). These results suggest that Nrf2 is a crucial antiosteoporotic factor whose suppression might be a general feature of the pathogenesis of OP.

### The Nrf2 promoter is hypermethylated accompanied by Dnmt alterations in osteoporotic femurs

**Fig. 2:**
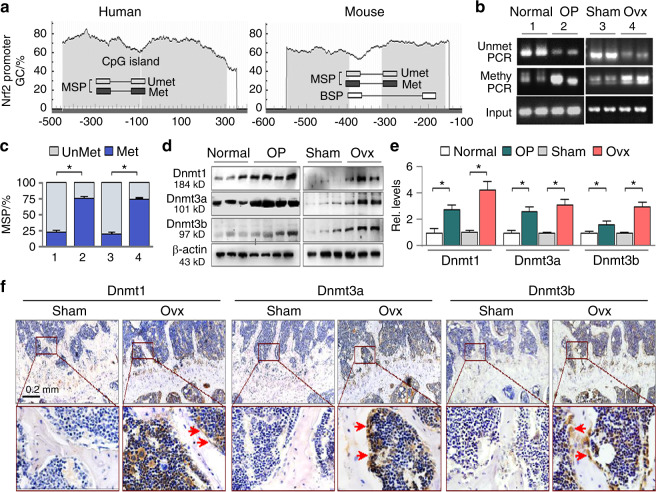
The Nrf2 promoter is hypermethylated in the femurs of osteoporotic patients and ovariectomized mice. **a** Schematic diagrams of human and murine Nrf2 promoters. The positions of CpG islands (gray area) and MSP/BSP primers are depicted relative to the transcription start site. **b** Representative agarose gel analyses of MSP products (methylated, unmethylated and input PCR) from the femurs of 4 control individuals and 3 patients with OP (the left panel) and the sham or ovariectomized mice (the right panel, 5 weeks after Ovx, 6 mice in each group, two representative samples are shown). **c** Quantifications of MSPs of Fig. 2b. Values are presented as percentage ± SEM of methylated/unmethylated PCR over total PCR products after adjustment with input controls from all human samples and experimental mice. *P < 0.05, one-way ANOVA. **d** Western blots of femoral Dnmt1, Dnmt3a, and Dnmt3b from human and mouse femurs (three randomly selected mouse samples are shown). **e** Quantifications of Fig. 2d. *P < 0.05, one-way ANOVA. **f** Representative mouse femur sections stained for Dnmt1, Dnmt3a, and Dnmt3b by immunohistochemistry (IHC). The lower panel images are the enlarged frames of the upper panel. The positively stained cells in brown are indicated by arrows

To explore the potential epigenetic mechanisms of aberrant DNA methylation that might account for Nrf2 suppression, we analyzed the Nrf2 promoter by the online software MethPrimer (http://www.urogene.org/methprimer). Both human and murine Nrf2 gene promoters are characterized by typical CpG islands located at the −450/300 (human) and −550/−120 regions (murine), as depicted in Fig. 2a (gray areas). We then examined the femoral DNA methylation status of Nrf2 promoters by MSP using methylated and unmethylated PCR primer sets designed by the software. The femoral samples from both patients with OP and ovariectomized mice exhibited increased Nrf2 promoter methylation compared to the healthy or sham controls (74.4% ± 3.76% versus 21.9% ± 3.6% for humans and 73.8% ± 2.73% versus 19.5% ± 2.9% for mice, respectively, *P* < 0.05). Figures 2b and 2c. To identify the potential upstream inducers of hypermethylation, we examined the major DNA methyltransferases and found that femoral Dnmt1, Dnmt3a and Dnmt3b were all upregulated in both the patients with OP and the ovariectomized mice (Fig. 2d and e), and immunohistochemistry (IHC) of the mouse femur sections confirmed the elevations (Fig. 2f). Taken together, these results suggest that Nrf2 suppression in femurs of both the patients and mice with OP is likely due to aberrant Dnmt1/3a/3b elevations and consequential Nrf2 promoter hypermethylation.

### Running exercise demethylates the Nrf2 promoter and inhibits aberrant femoral Dnmt expression

**Fig. 3:**
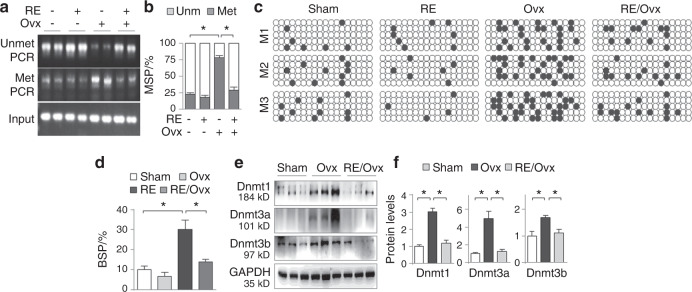
Running exercise demethylates the Nrf2 promoter and inhibits aberrant femoral Dnmt expression. Female ICR mice were treated and divided into the sham, Ovx, RE, and RE/Ovx groups (6 mice in each group) as described in the methods. **a** MSP assay. Representative agarose gel analysis of MSP products. Two samples from each group are shown. **b** Quantifications of MSP products in Fig. 3a. Values are presented as percentage changes of methylated/unmethylated PCR over total PCR products after adjustment with input controls, *P < 0.05, two-way ANOVA. **c** BSP assay. Three randomly selected mice (M1, M2, and M3) from each group were analyzed by BSP (the primer positions on the Nrf2 promoter are shown in Fig. 2a, right panel). The PCR products were cloned, and five clones from each PCR were sequenced. One box represents one mouse. Each row of dots in the box represents one single sequenced clone, and each dot represents one CpG site. Empty or dark dots indicate unmethylated or methylated CpGs, respectively. **d** Quantification of Fig. 3c. Data are presented as the mean percentage ± SEM of methylated CpGs over total CpGs in each group. *P < 0.05, two-way ANOVA. **e** Western blotting of mouse femoral Dnmt1, Dnmt3a and Dnmt3b. Three randomly selected samples from each group are shown. **f** Quantifications of Fig. 3e. ^*^P < 0.05, one-way ANOVA

Proper physical exercises are beneficial for maintaining bone health against osteoporosis.^17^ Recent studies report that physical exercises possess epigenetic DNA methylation modulating capacity,^11,12^ which might contribute to their osteoprotection against OP. To test this idea, we assigned half of the sham and the ovariectomized mice to daily 1-h treadmill running for a total of 4 weeks after 1 week of recovery from the surgery and examined the effects of RE on the Nrf2 methylation status in the mouse femurs. The results showed that the ovariectomized mice displayed increased Nrf2 promoter methylation in the femurs (78.38% ± 3% compared to 22.83% ± 2.05% in the healthy controls, *P* < 0.05). The ovariectomized mice that underwent 1 h of daily treadmill running showed a reduced level to 28.68% ± 5.3% (*P* < 0.05, Fig. 3a and b). To confirm these observations, we performed additional methylation analysis of the same region by bisulfate-specific PCR (BSP), the gold standard of methylation analysis. The results showed that CpG methylation was significantly increased in the ovariectomized mice from 10% ± 0.85% to 30% ± 2.14% (*P* < 0.05), whereas RE lowered it to 14.07% ± 0.57% (*P* < 0.05, Fig. 3c and d). More strikingly, RE also significantly mitigated the increases in Dnmt1, Dnmt3a and Dnmt3b (Fig. 3e and f), suggesting that RE might inhibit Nrf2 suppression by blocking Dnmt aberrations and subsequent Nrf2 promoter hypermethylation.

### Running exercise derepresses Nrf2 and protects the ovariectomized mice from osteoporosis

**Fig. 4:**
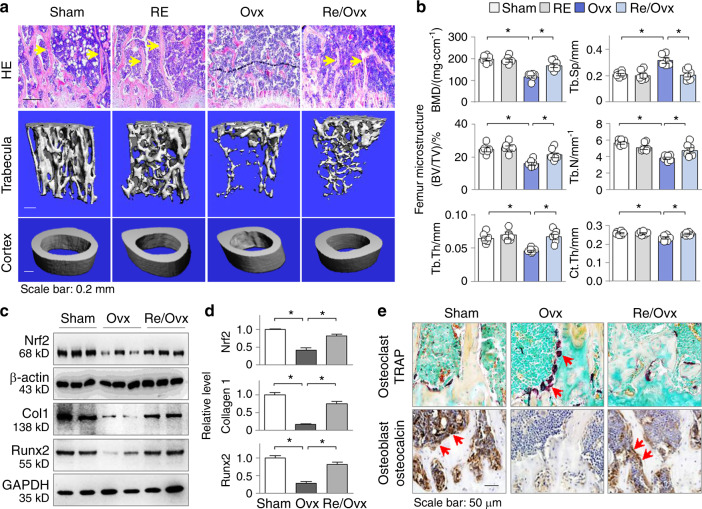
Running exercise derepresses Nrf2 and protects mice from osteoporosis. Female ICR mice were divided into the sham, RE, Ovx and RE/Ovx groups as described in the methods (6 mice in each group). **a** Representative photomicrographs of H&E-stained (the upper panel), micro-CT-scanned distal femoral trabecula (the middle panel) and cortex (the lower panel). **b** Three-dimensional microstructural analyses of distal femurs in Fig. 4a. Bone mineral density (BMD, mg·ccm^−1^), the ratio of bone volume to tissue volume (BV/TV), trabecular number (Tb.N), trabecular thickness (Tb.Th), trabecular separation (Tb.Sp) and cortical thickness (Ct.Th) were assessed. Data are presented as the mean ± SEM, *P < 0.05, two-way ANOVA. **c** Western blotting of Nrf2, collagen 1 (Col 1) and Runx2 from femur homogenates of the experimental mice. Two or three representative samples from each group are shown. **d** Quantification of Fig. 4c. Data are presented as the mean ± SEM of all experimental mice. *P < 0.05, one-way ANOVA. **e** Representative microphotographs of immunohistochemistry staining for TRAP and osteocalcin from mouse femur sections. The positively stained cells are indicated by arrows

Next, we wanted to determine whether RE corrections of the Dnmt alterations and Nrf2 promoter hypermethylation lead to Nrf2 recovery and protection against OP. The mice were divided into the sham, RE, Ovx and RE/Ovx groups (6 animals in each group). As anticipated, H&E staining of the femur sections showed that 1-h daily treadmill RE did not affect the normal femur histomorphology but mitigated the decreases in trabecular thickness and connectivity observed in the ovariectomized mice (Fig. 4a, upper panel). Micro-CT 3D analysis showed that the ovariectomized mice displayed typical osteoporotic alterations, such as reduced BMD, a lower trabecular bone volume versus tissue volume ratio (BV/TV), reduced trabecular bone number (Tb.N) and trabecular thickness (Tb.Th), increased trabecular bone separation (Tb.Sp) and reduced cortical thickness (Ct.Th, comparing groups 1 and 3 in Fig. 4a, the lower panel, and 4b). However, the RE effectively normalized the alterations (Fig. 4a, the lower panel, and b, comparing groups 4 and 3). Furthermore, femoral Nrf2 was suppressed in the ovariectomized mice, but RE significantly restored its level (Fig. 4c and d, upper panel). The osteoporotic femurs of the ovariectomized mice exhibited decreased levels of collagen 1 and the major osteoblast markers osteocalcin and Runx2 (Fig. 4c, d and e), as well as increased levels of the osteoclast marker TRAP (Fig. 4e). However, RE significantly corrected the abnormal expression, indicating that Nrf2 preservation by RE might exert an osteoprotective effect by inversely enhancing osteoblastogenesis and inhibiting osteoclastogenesis.

### Running exercise mitigates the suppression of antioxidant enzymes downstream of Nrf2

**Fig. 5:**
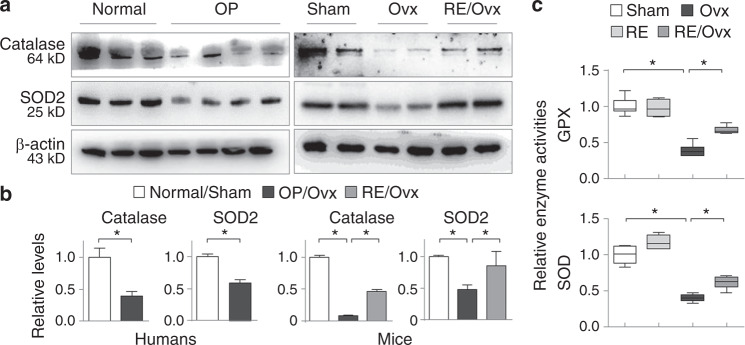
Running exercise mitigates the suppression of antioxidant enzymes. **a** Western blotting of femoral catalase and SOD2 from 3 healthy controls and 4 patients with OP, as well as the sham, ovariectomized and RE/ovariectomized mice (6 mice in each group, two randomly selected samples from each group are shown). **b** Quantifications of samples in Fig. 5a. *P < 0.05, one-way ANOVA. **c** The enzyme activities of GPX and SOD were examined from mouse femoral homogenates of the sham, RE, Ovx and RE/Ovx groups. Data are presented as the mean ± SEM of all experimental mice. *P < 0.05, two-way ANOVA

Nrf2 regulates oxidative stress by directly targeting and transactivating downstream antioxidant and detoxification enzymes, including catalase, SOD and GPX, which are critical for OP development;^28,30^ therefore, it is possible that osteoporotic Nrf2 deficiency might affect the expression of its downstream targets. Indeed, the osteoporotic femurs from both patients with OP and ovariectomized mice displayed reduced catalase and SOD2 proteins, direct Nrf2 targets and key antioxidant enzymes in bone.^31^ However, RE effectively alleviated the reductions in both enzymes in the ovariectomized mice, as shown by Western blotting (Fig. 5a and b). We also assessed the enzymatic activities of femoral GPX, another Nrf2 target, and total SOD using the respective commercial kits. The results indicated that ovariectomized mouse femurs exhibited reduced enzymatic activities of GPX and SOD compared to the controls (*P* < 0.05); however, RE significantly inhibited these reductions (*P* < 0.05, Fig. 5c). These results suggested that RE-mediated preservation of Nrf2 restores its transactivation of downstream antioxidative enzymes, which facilitates the removal of reactive oxygen species.

### Nrf2 is essential for the antiosteoporotic effects of running exercise

**Fig. 6:**
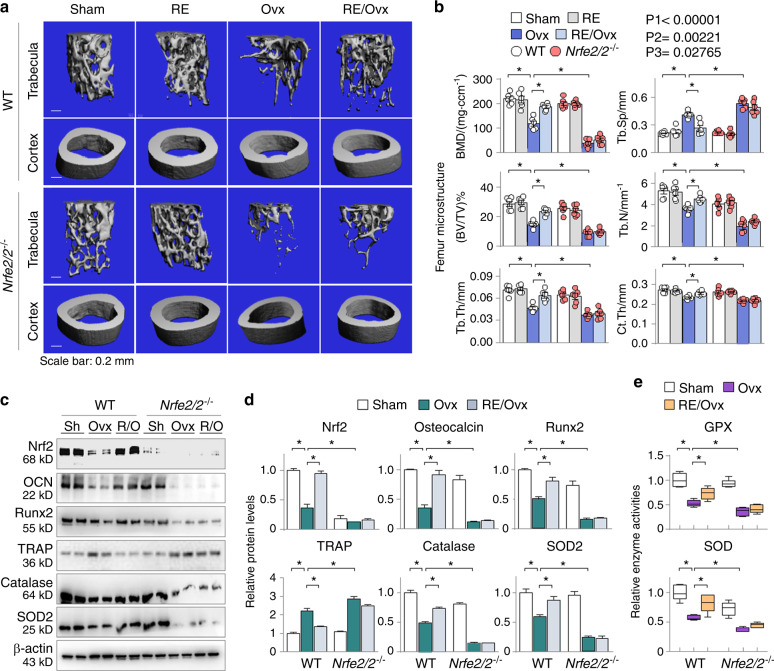
Nrf2 is essential for the antiosteoporotic effects of running exercise. *Nfe2l2*^−*/−*^ mice and the control littermates (WT) were subjected to the sham, RE, Ovx and RE/Ovx treatments as described in the methods (6 mice in each group). **a** Representative photomicrographs of micro-CT-scanned femoral trabeculae and cortex. **b** Quantitation of the samples in Fig. 6a. BMD (mg·ccm^−1^), the ratio of bone volume to tissue volume (BV/TV), trabecular number (Tb.N), trabecular thickness (Tb.Th), trabecular separation (Tb.Sp) and cortical thickness (Ct.Th) were assessed. *P < 0.05, three-way ANOVA with Tukey’s post hoc test. *P* values for the effect of genotype (P1), running intervention (P2), and the interaction between genotype and running intervention (P3) are indicated. **c** Western blotting of femoral Nrf2, osteocalcin (OCN), Runx2, TRAP, catalase and SOD2. Two representative samples from each group are shown. **d** Quantification of the samples in Fig. 6c. **e** The enzyme activities of GPX and SOD were examined in mouse femoral homogenates. **d** and **e** Data are presented as the mean ± SEM of all experimental mice. *P < 0.05, two-way ANOVA with Tukey’s post hoc test

To confirm the critical role of Nrf2 derepression by RE in OP, we compared the osteoprotective effects of RE between the *Nfe2l2*^*−/−*^ and control WT mice. Both the *Nfe2l2*^*−/−*^ mice with a substantial Nrf2 reduction (Fig. 6c, the top panel) and the WT mice were separately subgrouped into the sham, RE, Ovx, and RE/Ovx groups as before. As anticipated, the WT ovariectomized mice displayed marked femoral osteoporotic alterations in BMD, trabecular bone volume versus tissue volume (BV/TV), trabecular bone number (Tb.N), trabecular thickness (Tb.Th), trabecular bone separation (Tb.Sp), and cortical thickness (Ct.Th), as observed before in Fig. 4, and RE significantly improved these abnormalities (Fig. 6a, the upper panel, and b, comparing groups 4 and 3). The *Nfe2l2*^*−/−*^ sham mice exhibited a slight but not significant BMD reduction compared to that of the WT sham mice; however, they developed much worse OP alterations after Ovx (Fig. 6a, the lower panel, and b, comparing groups 7 and 3). Notably, the OP-protective effects of RE observed in the WT mice were largely diminished in the *Nfe2l2*^*−/−*^ mice (Fig. 6a, the lower panel, and b, comparing groups 8 and 7). We also calculated the effects of group and group interaction and found that BMD was greatly influenced by the Nrf2 genotype (*P*1 < 0.000 01), RE intervention (*P*2 = 0.002 21) and the interaction between the Nrf2 genotype and RE intervention (*P*3 = 0.027 65). Similarly, RE significantly reduced the abnormal expression of Nrf2, Runx2, osteocalcin (OCN), TRAP, catalase and SOD2 (Fig. 6c and d, the left panels) and improved the enzymatic activities of GPX and total SOD (Fig. 6e, the left panel) in the WT mice, but the beneficial effects were largely diminished in the *Nfe2l2*^*−/−*^ mice (Fig. 6c, d and e, the right panels), indicating that Nrf2 derepression is essential for the osteoprotective effects of RE in the ovariectomized mice. Because RE inversely regulates the marker expression of both osteoblasts (Runx2, osteocalcin) and osteoclasts (TRAP) in an Nrf2-dependent manner (Fig. 6c and d), these results also indicate that RE-induced Nrf2 preservation beneficially affects both osteoblasts and osteoclasts.

## Discussion

**Fig. 7:**
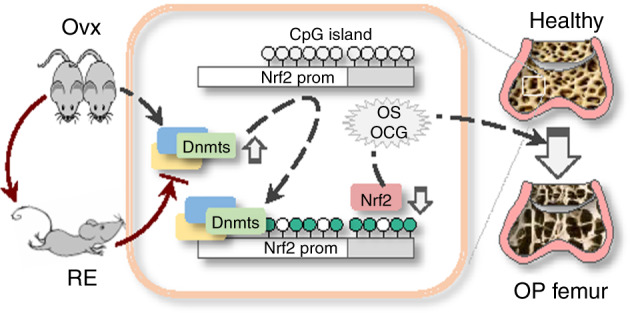
A schematic diagram of mouse running exercise, Nrf2 promoter demethylation and protection against osteoporosis (OP). Ovariectomized mice display increased femoral Dnmt1/3a/3b (Dnmts), Nrf2 promoter hypermethylation and Nrf2 suppression, which promote oxidative stress (OS), osteoclastogenesis (OCG) and OP (dashed line). Running exercise (RE, solid line) normalizes Dnmt aberrations, resulting in Nrf2 promoter demethylation, Nrf2 recovery and reduced femoral osteoporotic pathologies

In this study, we report several novel findings that help elucidate the epigenetic pathogenesis of OP and the antiosteoporotic effects of RE. We revealed that the *Nfef2l2* deficient mice develop more severe OP after Ovx than the WT mice and that Nrf2 is markedly repressed in the osteoporotic femurs of both patients with OP and ovariectomized mice. Nrf2 repression is likely due to aberrant elevations in Dnmt1, Dnmt3a and Dnmt3b and consequential Nrf2 promoter hypermethylation. We further showed that 1 h of daily treadmill running of the ovariectomized mice effectively reversed epigenetic alterations and recovered Nrf2 loss, and Nrf2-dependently protected against femur BMD loss (Fig. 7). These results uncover an important epigenetic feature of the pathogenesis of OP that links the epigenetic modulatory capacity of physical exercise to its antiosteoporotic function.

Proper physical exercises including both weight-bearing exercise, such as walking, stair climbing, jogging, and strength/resistance exercise exemplified by weight lifting stimulate significant osteogenic responses that preserve bone strength and integration beneficial for patients with OP, especially postmenopausal women.^18,32^ Physical exercises presumably confer osteoprotective effects by generating mechanical loadings on the affected muscles and bones;^33^ however, the precise osteogenic cellular processes that transform the mechanical forces to osteoprotection against OP are still unclear. Epigenetic modifications are emerging as a new mechanism of altered gene expression in the pathogenesis of OP.^4,5^ Previous studies have reported altered methylation modifications of ALPL (alkaline phosphatase),^34^ SOST (sclerostin),^35^ and RANKL/OPG (osteoprotegerin),^36,37^ as well as several genes in the Wnt pathway^38^ that are closely related to osteopenia and OP. However, it is unclear whether the alterations are the causes or the results of OP progression. Our results showed that RE effectively mitigates aberrant Dnmt1/Dnmt3a/Dnmt3b elevations, the associated Nrf2 promoter hypermethylation and Nrf2 suppression and that Nrf2 improves femur BMD and trabecular microstructure (Figs. 2, 3, 4 and 6). Therefore, we propose that Nrf2 is a key gene of DNA methylation modification that promotes OP and that RE exerts its antiosteoporotic effects at least in part via epigenetic Nrf2 preservation.

Adequate balance of redox responses by Nrf2 is critical for bone health.^21,39^ Weaker or deficient oxidative stress responses with excessive reactive oxygen species (ROS) promote osteoclastogenesis^40^ and inhibit osteoblastogenesis,^19^ resulting in increased bone absorption and OP.^41^ Nrf2 induces antioxidative stress responses by transactivating major detoxifying enzymes causally related to OP. SOD, catalase and GPX are direct targets of Nrf2 and first-line defense antioxidants.^42^ SOD catalytically converts superoxide radicals (^∗^O_2_^-^) to hydrogen peroxide (H_2_O_2_) and molecular oxygen (O_2_). Catalase further catalyzes the reduction of H_2_O_2_ to water and O_2_, completing the detoxification process. In mitochondria, where catalase is absent, the later reduction step is carried out by GPX.^42^ Therefore, RE-induced Nrf2 derepression and the subsequent reactivation of SOD, catalase and GPX presumably reduce excessive oxidants and improve BMD. In addition, Nrf2 possesses broad biological activities directly affecting cellular processes of osteogenesis.^21,23^ For example, Nrf2 can prevent osteocyte death by regulating cell autophagy^43,44^ and inversely and beneficially affect apoptosis of osteoblasts and osteoclasts.^45,46^ We indeed observed that RE normalized the reductions in the antioxidant enzymes catalase and SOD and the adverse expression of osteoblast and osteoclast markers in an Nrf2-dependent manner (see Fig. 6). These results indicate that Nrf2 preservation by RE provides sufficient osteoprotection against OP by beneficially regulating multiple cell types and pathological cellular processes.

Information regarding the upstream events that cause DNA methylation alterations critical for OP development remains inconclusive. A previous study reported that Dnmt1 expression was upregulated in the femoral tissue of rats with disuse osteoporosis. Local intramedullary injection of Dnmt1 siRNA abrogated the femoral microstructure declines.^47^ Another study found that Dnmt3b was increased in femoral bone marrow stromal cells, the precursors of osteoblasts, isolated from hindlimb-unloaded mice with OP. Treatment with the Dnmt3b-selective inhibitor neomycin A partly rescued bone loss.^48^ More pertinently, in ovariectomized mice, administration of the general DNA demethylating agent decitabine (5-aza-2’-deoxycytidine) or the Dnmt3a-selective inhibitor theaflavin-3,30-digallate (TF-3) reduced osteoclast activity and improved BMD, respectively.^9,49^ These results suggested that increased Dnmt abundance/activities occur in multiple bone cells and drive the initiation or progression of OP. We screened bioactive Dnmt1, Dnmt3a and Dnmt3b and found marked elevations in all three Dnmts in the femurs of both patients with OP and ovariectomized mice that correlated with Nrf2 promoter hypermethylation and Nrf2 repression. More impressively, RE inhibits the upregulation of all three Dnmts and the Nrf2 promoter hypermethylation, resulting in the recovery of Nrf2 and its downstream antioxidant enzymes and improving femur BMD and microstructure. These findings provide strong evidence that aberrant Dnmt1/3a/3b elevations are the major upstream causal factors of Nrf2 repression that contribute to the epigenetic pathogenesis of OP.

In summary, the results from our study revealed that Nrf2 repression due to aberrant Dnmt elevations and the associated promoter hypermethylation contributes significantly to the epigenetic development of OP and that RE effectively corrects epigenetic abnormalities and the pathogenesis of OP in mice, which have potential clinical implications. Aberrant epigenetic modifications are important mechanisms through which environmental and stochastic stressors promote numerous pathological processes, including OP;^3^ therefore, people with epigenetic Nrf2 suppression due to past adverse environmental exposures, such as air pollution, a poor diet, drug use or illnesses, might be prone to or develop worse OP. In addition, because a combination of weight-bearing aerobic exercises and strength/resistance exercises reportedly exhibit better osteogenic effects,^10^ we speculate that RE/jogging together with other strength/resistance exercises might have much more effective epigenetic modulating capacities that prevent or retard the onset and progression of OP.

## Materials and methods

### Animal studies

The use of animals and the experimental protocols were approved by the Animal Care Committee of Nanjing University in accordance with the Institutional Animal Care and Use guidelines. Female ICR mice (from the Model Animal Research Center of Nanjing University), Nrf2 gene knockout mice (*Nfe2l2*^−/−^, with permission from Dr. Yamamoto Masayuki) and WT littermates reported previously^50^ were housed in an onsite animal facility with free access to water and diet under a 12 h light/dark cycle.

A mouse model of OP was established with a 5-week Ovx protocol.^29^ Female mice of approximately 10–12 weeks old were divided into (I) the sham surgery, (II) running exercise (RE), (III) Ovx, or (IV) RE/Ovx groups (at least 6 mice in each group). Experimental mice were anesthetized with isoflurane, and Ovx was performed with aseptic surgery following a previously described procedure.^49^ Briefly, the bilateral ovaries of the ovariectomized mice were removed through a midline incision of the skin and flank incisions of the peritoneum. The skin incision was then closed with metallic clips. Mice subjected to sham operation were processed similarly without ovary removal. After one week of recovery from the surgery, the mice in the RE and RE/Ovx groups were placed on a mouse treadmill at a low speed (10 m·min^−1^) for 1 h every day for a total of 4 weeks. On completion, the mice were sacrificed, and the mouse hind legs were isolated and frozen at −80 °C for further analyses. Six young (3 months) and 6 old (25 months) ICR male mice were also used for comparison of femoral Nrf2 expression.

### Bone micro-CT analysis

For trabecular microstructure analysis, mouse left femurs were fixed with 4% paraformaldehyde for 24 h, and the mid-diaphysis and distal metaphysis of femurs were selected as the region of interest and scanned with a micro-CT scanner (Scanco Medical, Bruettisellen, Switzerland). The scanner was set at 55 kV, 145 μA and 15.6 μm voxel size with 250 ms integration time. A total of 100 slices from each sample were used to analyze the trabecular microarchitectures at the distal metaphyses 780 μm from the growth plate. Cross sections and three dimensional (3D) trabecular images were acquired, and the sectional trabeculae over the total area, bone mineral density [BMD, in hydroxyapatite (HA) concentration, mg·ccm^−1^], ratio of bone volume to tissue volume (BV/TV), trabecular number (Tb.N), trabecular thickness (Tb.Th) and trabecular separation (Tb.Sp) were calculated. The femoral cortical thickness (Ct.Th) was measured by analyzing 50 slices of the mid-diaphysis for each sample.

### Human samples

Human samples were collected from patients receiving joint replacement at Nanjing Drum Tower Hospital of Nanjing University Medical School. OP is defined as a T score of ≤−2.5, and a T score of ≥−1 is considered normal bone density according to the National Osteoporosis Foundation.^51^ Osteoporotic femurs were obtained from 4 female patients with osteoarthritis (68–75 years old) with an average bone densitometry T score of −3.49 (−2.6, −3.24, −5.46, and −2.67), as determined by dual energy X-ray absorptiometry. The control non-OP femurs were from three age-matched patients (49–71 years old) with an average T score of −0.67 (−0.98, −0.64, and −0.4). Two non-OP patients suffered from osteoarthritis, and one was diagnosed with congenital hip dysplasia. The samples were stored at −80 °C before further protein, histology and MSP analyses. The study was approved by the ethics committee of Nanjing Drum Tower Hospital (2016-200-01), and written informed consent was received from all subjects. Patients or members of the public are not involved in the design, conduct, reporting, or dissemination plans of the research.

### Histological and immunohistochemical (IHC) staining

Human and murine knee joint sections were processed with hematoxylin and eosin (H&E), TRAP (tartrate-resistant acid phosphatase), or IHC staining essentially as described before.^52^ For IHC staining, section slides were incubated overnight at 4 °C with primary antibodies against Nrf2, Dnmt1, Dnmt3a, Dnmt3b, or osteocalcin and then with HRP-conjugated secondary antibodies. Afterward, the slides were processed with a DAB horseradish peroxidase color development kit (Beyotime Biotech, USA) and counterstained with hematin as previously described.^50^ TRAP staining was performed with a commercial kit according to the manufacturer’s instructions (Sigma, 387A-1KT) and counterstained with methyl green.

### Western blot assay of protein expression

Western blot assays of femur homogenates from human or murine samples under various conditions were performed essentially as before.^50^ The primary antibodies used were Nrf2 (Cell Signaling Technology, Boston, USA); Runx2, TRAP and catalase (Abcam, Cambridge, USA); Dnmt1 (Novus, USA); collagen 1, Dnmt3a and Dnmt3b (ABclonal, Wuhan, China); and osteocalcin, SOD2, GAPDH, and β-actin (Santa Cruz, USA). Western blots were visualized with an ECL plus Western blotting detection system (Yifeixue Biotech, Nanjing, China), and the protein quantities were analyzed by ImageJ software.

### Methylation-specific PCR (MSP) and bisulfite-sequencing PCR (BSP)

Analysis of CpG islands and primer design of human and mouse Nrf2 promoters for methylation-specific PCR (MSP) and bisulfite-sequencing PCR (BSP) were performed with the online MetPrimer software (http://www.urogene.org/methprimer).^53^ MSP was performed with genomic DNA from human and mouse femur samples following a previously established protocol.^54^ To analyze methylation of the human Nrf2 promoter by MSP, we used the methylated forward primer TAAAAATGTGTTTAGTTACGGGGTC (−289/−265) and the reverse primer CTCAAAACTACCAAAAAATAATCCG (−98/−123) and the unmethylated forward primer AAAAATGTGTTTAGTTATGGGGTTG (−288/−264) and the reverse primer CAAAACTACCAAAAAATAATCCAAA (−100/−125). The PCR control for input DNA (Input) was performed with the forward primer CCAACTCCAAATCCCCTCTCTAT and the reverse primer TGATTAATTTAGATTGGGTTTAGAGAAGGA.

For MSP analysis of the mouse Nrf2 promoter, we used the methylated forward primer GTTTTAAAGA GTTAGGGTTGGAGC (−398/−375) and the reverse primer AAATCAAATAAACTAAAATCGCACG (−256/−280) and the unmethylated forward primer TGTTTTAAAGAGTTAGGGTTGGAGT (−399/−375) and the reverse primer ATCAAATAAACTAAAATCACACAAA (−257/−282). The forward primer TAGTTTTAGGAAGGTAAAGGGAGTG and the reverse primer AAATCCCAAAAAAAACACAACAAA were used as the internal controls. The PCR products were analyzed on a 2% agarose gel and visualized under ultraviolet light, and densitometric analysis was performed using ImageJ software. The relative levels of PCR products were first normalized with input PCR and then presented as the ratio of methylated or unmethylated PCR over total PCR products.

Bisulfite-sequencing PCR (BSP) analysis of the mouse Nrf2 promoter was carried out following a previously established laboratory protocol.^55^ In brief, bisulfite-treated genomic DNA of mouse femurs was amplified by PCR with the BSP forward primer TGTTTTAAAGAGTTAGGGTTGGAG (−399/−376) and the reverse primer AAAACCAATAAAAAAAACAAAAACC (−133/−157), with amplification of the same CpG island as MSP. The amplified PCR products were gel-purified with a PCR Product Purification Kit (Sangon Biotech, China) and cloned into the pGEM-TEasy-vector system (Promega, USA). Five colonies from each PCR were randomly chosen for sequencing, and the percentages of methylated cytosines over total cytosines within the cloned fragment were calculated.

### Measurement of antioxidant enzyme activities

The enzymatic activities of femoral GPX and total SOD were measured with commercial detection kits (Beyotime Institute of Biotechnology, China) following the manufacturer’s instructions. GPX activities were determined by a two-step enzymatic reaction, in which GPX first catalyzes GSH (glutathione) to produce oxidized glutathione (GSSG), and then, a fixed amount of NADPH (nicotinamide adenine dinucleotide phosphate) was consumed when glutathione reductase converts GSSG to GSH. The reduction in NADPH measured at 340 nm was lineally correlated with GPX activity. SOD enzyme activities were determined by measuring formazan dye at 450 nm that was converted from tetrazolium by total SOD. The enzyme activities are presented as relative fold changes compared to the controls.

### Statistical analysis

Statistical analyses were conducted using SPSS ver. 21.0 software. The normal distributions of the data and the assumptions of homogeneity of variances were assessed by the Shapiro-Wilk test and Levene’s test. Data were analyzed by 1- or 2-way ANOVA for one- or two-factor experiments or 3-way ANOVA for three-factor experiments followed by Tukey’s post hoc test for effects of group interactions and are presented as the mean ± SEM (standard error of mean). *P* < 0.05 was defined as statistically significant.

## Supplementary Information

Supplementary Information

Supplementary Information

Supplementary Information

Supplementary Information

## Supplementary Information

**Supplementary information** The online version of this article (10.1038/s41413-020-00128-8) contains supplementary material, which is available to authorized users.
