# Supplementary figures and images for: Nrf2 epigenetic derepression induced by running exercise protects against osteoporosis
Source: Bone Res. 2021 Feb 26;9:15. doi: 10.1038/s41413-020-00128-8 (PMC7910611; doi:10.1038/s41413-020-00128-8)

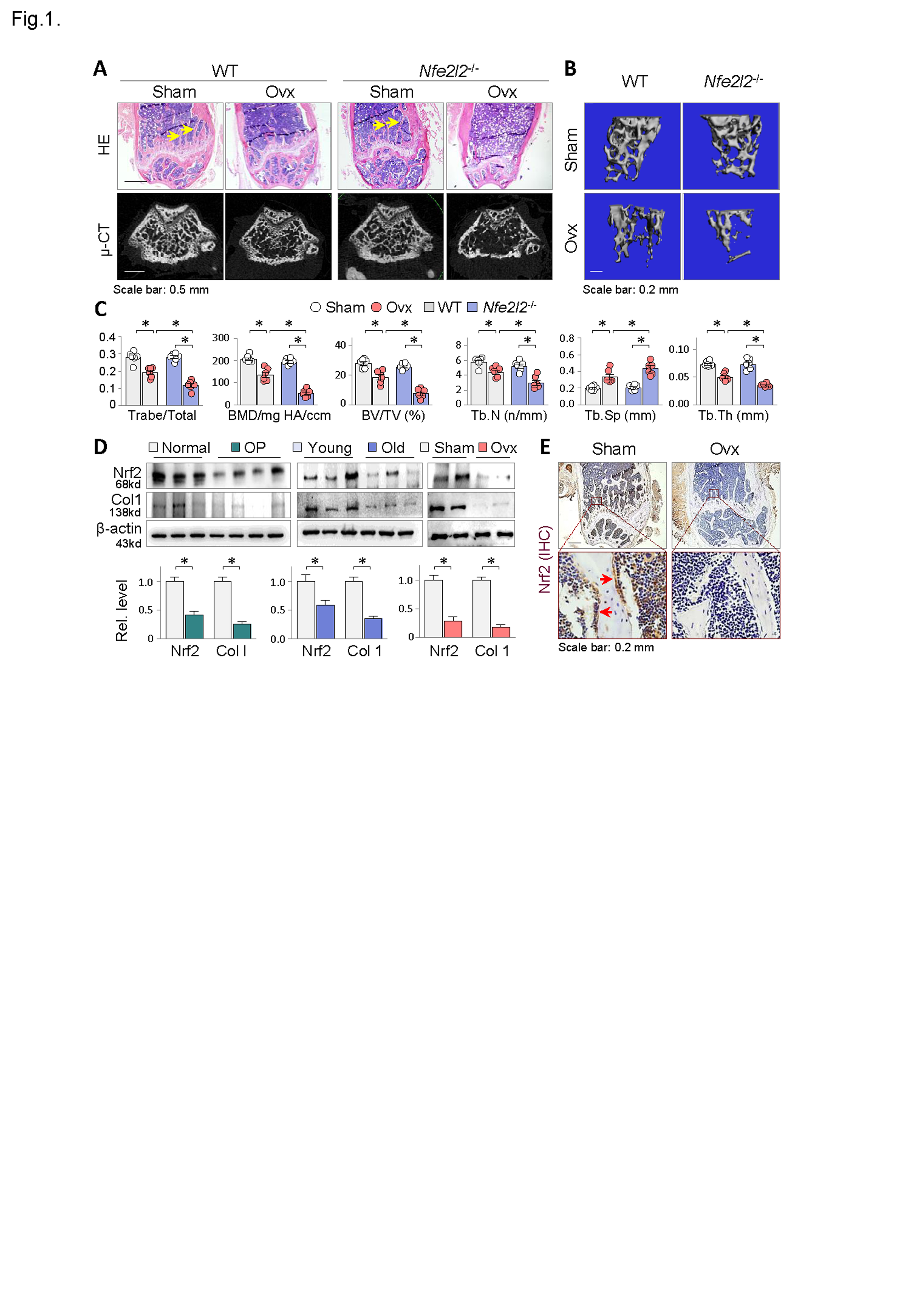

Supplement: Supplementary file 1 — Supplementary Information [file 41413_2020_128_MOESM1_ESM.tif]

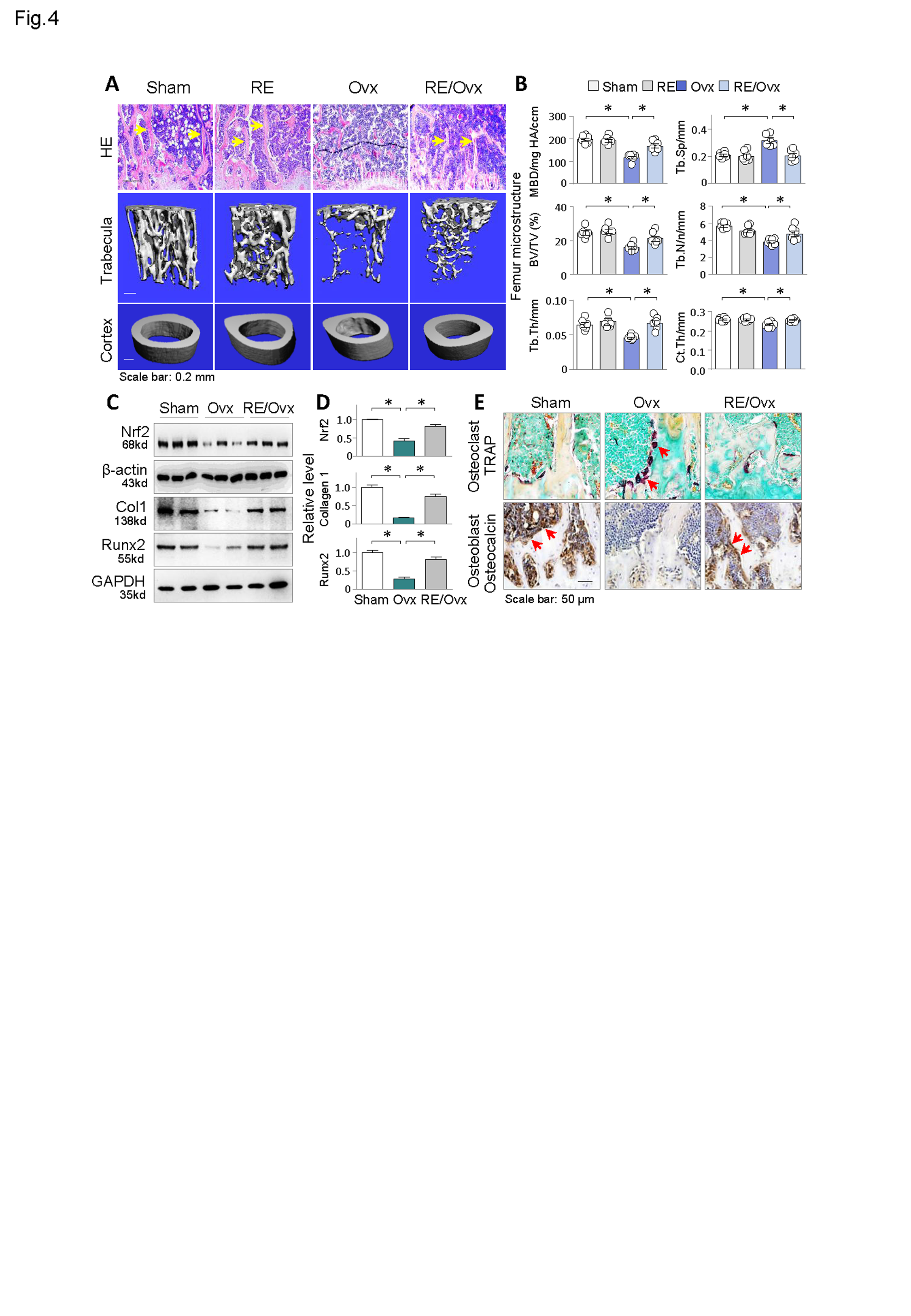

Supplement: Supplementary file 2 — Supplementary Information [file 41413_2020_128_MOESM2_ESM.tif]

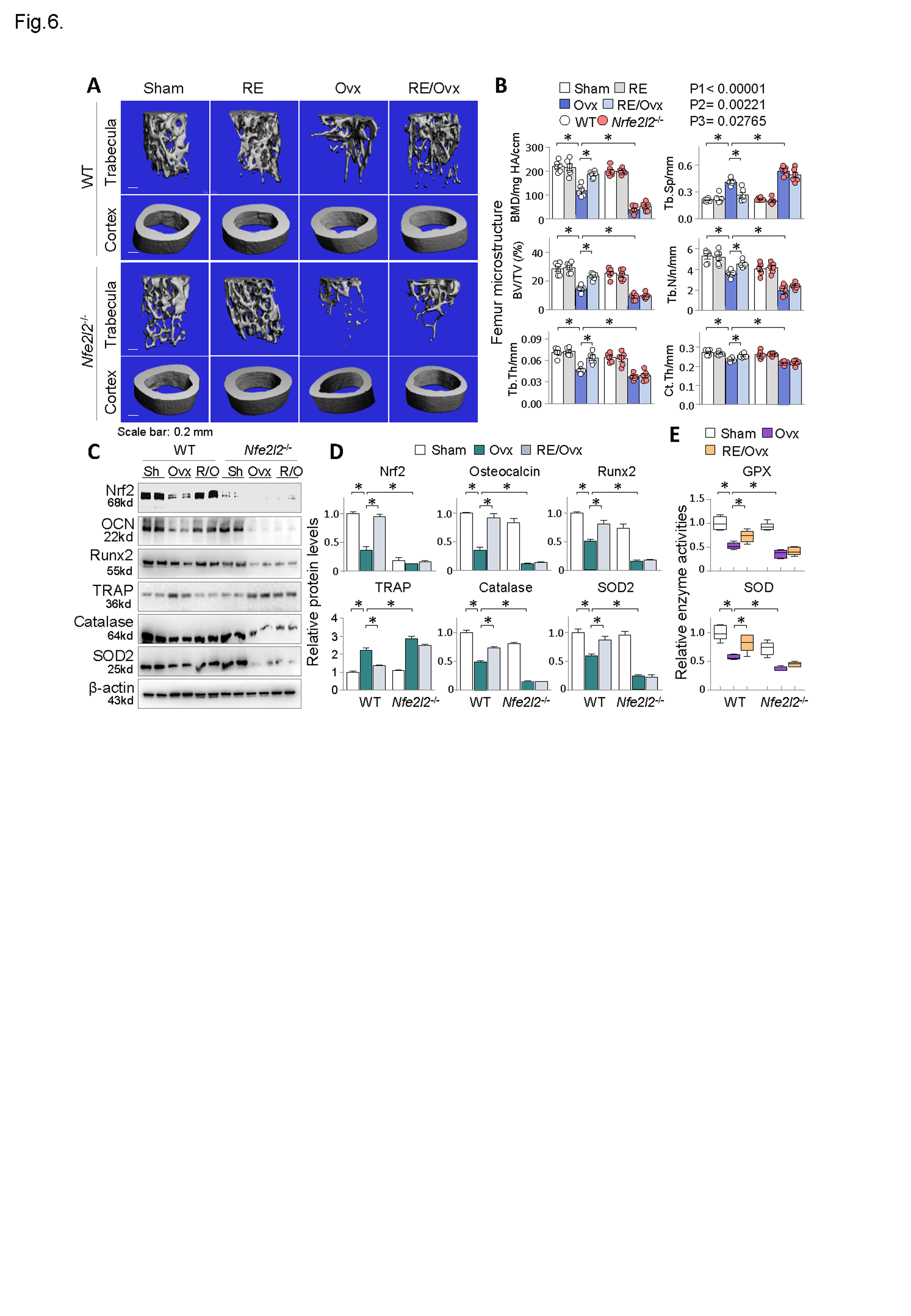

Supplement: Supplementary file 3 — Supplementary Information [file 41413_2020_128_MOESM3_ESM.tif]

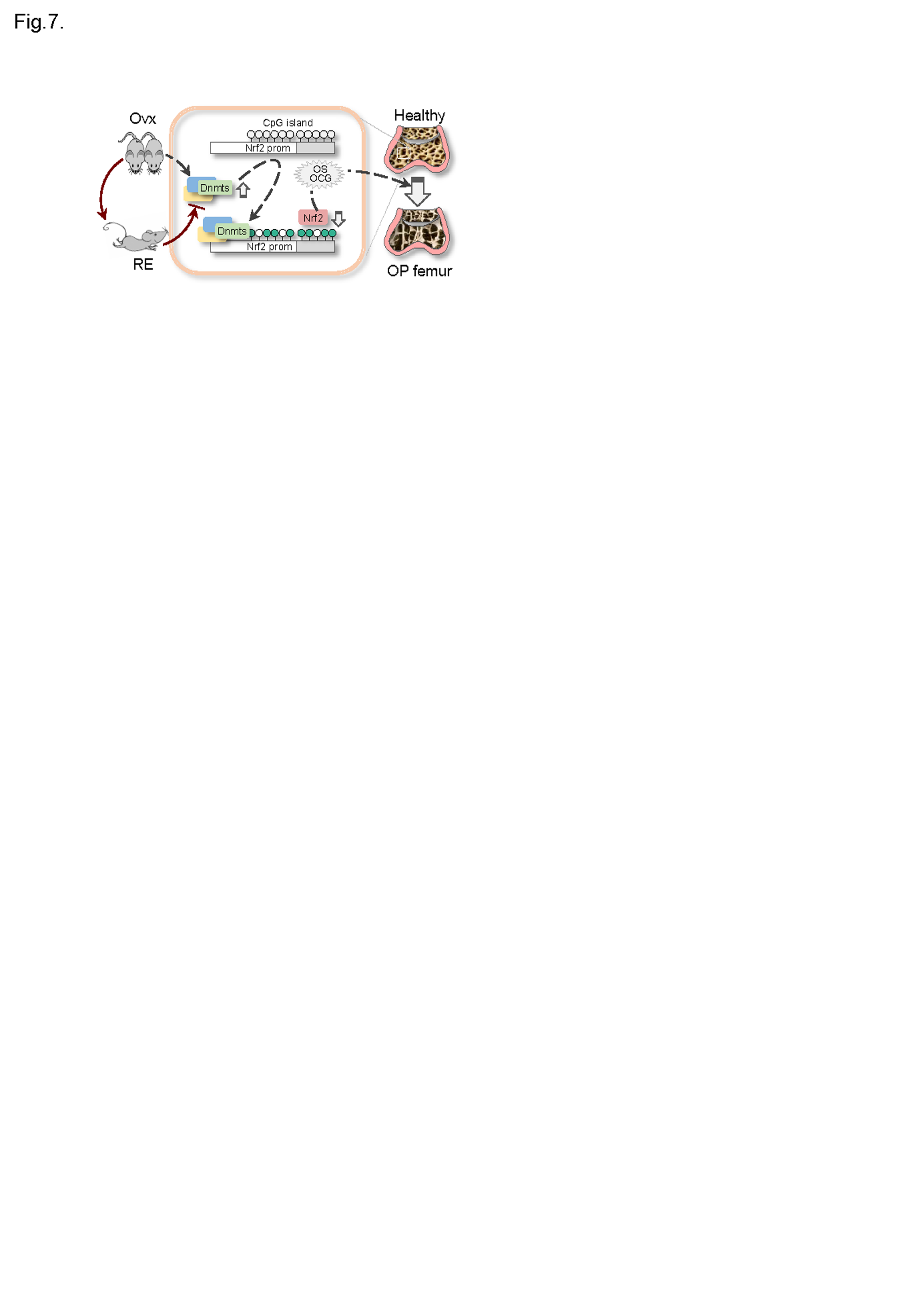

Supplement: Supplementary file 4 — Supplementary Information [file 41413_2020_128_MOESM4_ESM.tif]
